# Brillouin scattering in multi-core optical fibers for sensing applications

**DOI:** 10.1038/srep11388

**Published:** 2015-06-13

**Authors:** Yosuke Mizuno, Neisei Hayashi, Hiroki Tanaka, Yuji Wada, Kentaro Nakamura

**Affiliations:** 1 Precision and Intelligence Laboratory, Tokyo Institute of Technology, 4259 Nagatsuta-cho, Midori-ku, Yokohama 226-8503, Japan; 2 Faculty of Science and Technology, Seikei University, 3-3-1 Kichijoji Kitamachi, Musashino-shi, Tokyo 180-8633, Japan

## Abstract

We measure the Brillouin gain spectra in two cores (the central core and one of the outer cores) of a ~3-m-long, silica-based, 7-core multi-core fiber (MCF) with incident light of 1.55 μm wavelength, and investigate the Brillouin frequency shift (BFS) and its dependence on strain and temperature. The BFSs of both the cores are ~10.92 GHz, and the strain- and temperature-dependence coefficients of the BFS in the central core are 484.8 MHz/% and 1.08 MHz/°C, respectively, whereas those in the outer core are 516.9 MHz/% and 1.03 MHz/°C. All of these values are not largely different from those in a silica single-mode fiber, which is expected because the cores are basically composed of the same material (silica). We then analyze the difference in structural deformation between the two cores when strain is applied to the fiber, and show that it does not explain the difference in the BFS dependence of strain in this case. The future prospect on distributed strain and temperature sensing based on Brillouin scattering in MCFs is finally presented.

There is a growing demand for optical fibers with high transmission capacities that can slake our seemingly insatiable appetite for data. Although the spectral efficiency of these fibers has been extensively enhanced by using a variety of techniques^1^, the information-transmission capacity of a standard silica-based single-mode fiber (SMF) is approaching its limit because of the nonlinear effects of optical fibers^2^,^3^,^4^,^5^,^6^. A recent solution to this ever-increasing demand is space-division multiplexing based on multi-core fibers (MCFs)^7^,^8^,^9^,^10^,^11^,^12^,^13^,^14^,^15^. By exploiting such multiple cores, researchers have been drastically enhancing the transmission capacity delivered over a single fiber.

In the meantime, Brillouin scattering in silica SMFs has attracted considerable interest^16^,^17^,^18^,^19^ and has been applied to a number of devices and systems, including distributed strain and temperature sensors^20^,^21^,^22^,^23^,^24^. To improve the performance of these devices, Brillouin scattering properties in various special fibers have been investigated, and some of them have already been practically applied. Such special fibers include tellurite glass fibers^25^,^26^, chalcogenide glass fibers^27^,^28^, bismuth-oxide glass fibers^26^,^29^, photonic crystal fibers (PCFs)^30^, rare-earth-doped glass fibers (REDFs)^31^,^32^, polymethyl methacrylate (PMMA)-based polymer optical fibers (POFs)^33^, and perfluorinated graded-index (PFGI) POFs^34^,^35^. Each special fiber has its own unique advantage; for instance, the Brillouin-scattered Stokes powers in tellurite and chalcogenide fibers are much higher than those in silica SMFs, owing to their large Brillouin gain coefficients^25^,^26^,^27^,^28^; the Stokes powers in REDFs can be adjusted by controlling the pumping light power (at 980 nm in erbium-doped fibers (EDFs))^32^,^36^; and Brillouin scattering in POFs is potentially applicable to high-sensitivity temperature measurement^35^ as well as large-strain measurement^37^. Similarly, if Brillouin-scattered signals from multiple cores of an MCF can be simultaneously exploited, a discriminative measurement of strain and temperature^38^,^39^,^40^,^41^ will be feasible by using a single fiber, as discussed below. To the best of our knowledge, the Brillouin properties in MCFs have not been experimentally investigated yet. Therefore, clarifying these properties is the first step to explore their potentials in practical applications.

In this paper, the Brillouin gain spectra (BGS) in two cores (the central core and one of the outer cores) of a ~3-m-long, silica-based, 7-core MCF are measured at 1.55 μm, and the Brillouin frequency shift (BFS) and its dependence on strain and temperature are investigated. The BFSs of both the cores are ~10.92 GHz, which are higher than that of a standard silica SMF by over 50 MHz. The strain- and temperature-dependence coefficients of the BFS in the central core are 484.8 MHz/% and 1.08 MHz/°C, respectively, whereas those in the outer core are 516.9 MHz/% and 1.03 MHz/°C. All of these coefficients are nearly identical to the values in a silica SMF, as is expected considering that the cores are basically composed of the same material, i.e., silica. Subsequently, using finite element analysis (FEA), the difference in applied stress between the two cores when strain is applied to the fiber is calculated to be insufficiently large to cause the difference in the BFS dependence of strain. The future vision for MCF-based Brillouin sensors is finally discussed.

## Results

### Principle

Owing to an interaction with acoustic phonons, a light beam propagating through an optical fiber is backscattered, generating so-called Stokes light. This nonlinear process is known as Brillouin scattering^16^, where the central frequency of the Stokes light spectrum (called the BGS) becomes lower than that of the incident light. The amount of this frequency shift is referred to as the BFS, which is, in optical fibers, given as^16^
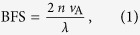
where *n* is the refractive index, *v*_A_ is the acoustic velocity in the fiber core, and *λ* is the wavelength of the incident light. As *n* and *v*_A_ are dependent on strain and temperature, the BFS also exhibits strain and temperature dependence, which serves as the operating principle of strain/temperature sensors. To date, the BFS dependence on strain and temperature has been investigated in a variety of special fibers. Table 1 summarizes the BFS and its dependence on strain and temperature, along with the refractive index, in silica SMFs^17^,^18^, tellurite fibers^25^,^26^, chalcogenide fibers^27^, bismuth-oxide fibers^26^,^29^, germanium-doped PCFs (main peak only)^30^, REDFs (erbium-31, neodymium-32, and thulium-doped)^32^, and PFGI-POFs^35^. Supposing that the wavelength of the incident light is 1.55 μm and that *n* is not dependent on the wavelength, all the values in Table 1 have been calculated using Eq. (1). As can be seen, the BFS and its strain and temperature dependence drastically vary depending on the fiber materials and structures, which is extremely important in considering the applications of Brillouin scattering in MCFs.

### Experimental setup

**Figure 1:**
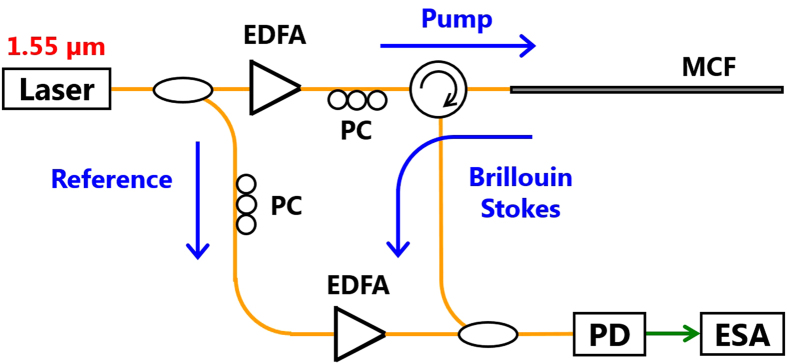
Schematic of the experimental setup for Brillouin measurement.

The experimental setup for investigating the BFS dependence on strain and temperature in the MCF is depicted in Fig. 1, where self-heterodyne detection was used to observe the BGS with a high frequency resolution^34^. The output of a laser diode at 1.55 μm was divided into two, one of which was amplified with an erbium-doped fiber amplifier (EDFA) to 14 dBm and used as reference light. The other was also amplified with another EDFA to 30 dBm and injected into the MCF as pump light. The backscattered Brillouin Stokes light was then coupled with the reference light. The optical beat signal was then converted into an electrical signal with a photo detector (PD), and observed with an electrical spectrum analyzer (ESA) as BGS. Each BGS measurement was performed 20 times, with the average being calculated thereafter. The relative polarization state between the Stokes light and the reference light was adjusted with polarization controllers (PCs). The room temperature was ~28 °C.

**Figure 2:**
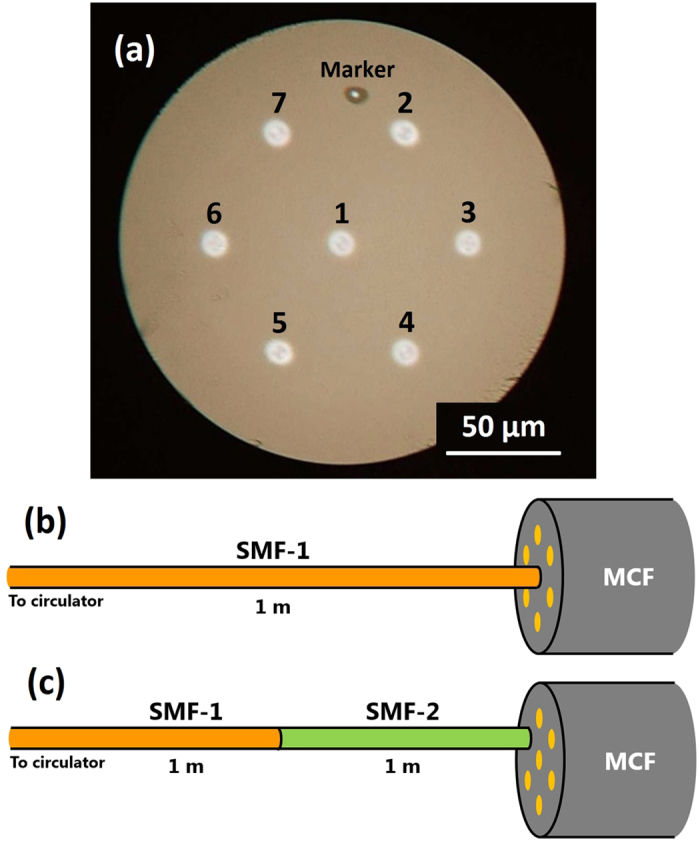
(**a**) Cross-sectional micrograph of the 7-core MCF. Structures of the fiber under test for detecting Brillouin scattering in (**b**) the central core and (**c**) one of the outer cores of the MCF.

We employed a ~3-m-long, silica-based, 7-core MCF as a fiber under test^11^, the cross-sectional micrograph of which is shown in Fig. 2(a). The cladding diameter was 197.0 μm, and the core pitch was, on average, 56.0 μm. At 1.55 μm, the mode-field diameter (MFD) and propagation loss of each core were 11.2 ± 0.1 μm and 0.198 ± 0.011 dB/km, respectively. When the Stokes light returned from the central core (#1 in Fig. 2(a)) of the MCF was detected, as shown in Fig. 2(b), one end of the MCF was spliced to a ~1-m-long silica SMF (termed as SMF-1), which was connected to a circulator using an arc-fusion splicer (central cores were automatically aligned), and the other end of the MCF was cut on an angle and immersed into matching oil (*n *= 1.46) to suppress the Fresnel reflection. In contrast, when the Stokes light returned from one of the outer cores (#2 in Fig. 2(a)) of the MCF was detected, as shown in Fig. 2(c), the outer core at one end of the MCF was spliced to one end of a ~1-m-long silica SMF (termed as SMF-2; manufactured by a company different from that of the SMF-1) using an arc-fusion splicer (accurate core alignment was required). The other end of the SMF-2 was spliced to a ~1-m-long SMF-1. The Fresnel reflection at the other end of the MCF was suppressed in the same way. Strain was applied to the whole length of the MCF, two parts near the ends of which were fixed on translation stages by epoxy glue to avoid slipping. The temperature of the entire MCF was manipulated via an external heater.

### Central-core characterization

**Figure 3:**
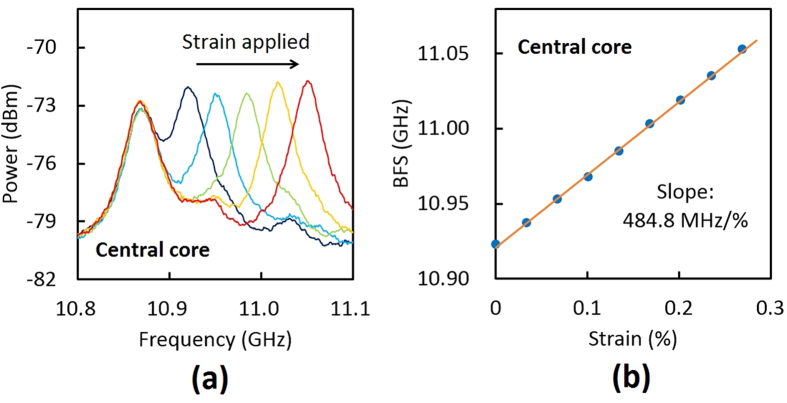
(**a**) BGS dependence on strain (0, 0.067, 0.135, 0.202, and 0.270%) and (**b**) BFS dependence on strain in the central core of the MCF.

First, the experimental results on the central core of the MCF are presented. The blue curve in Fig. 3(a) shows the BGS measured at room temperature. Two clear peaks (corresponding to BFSs) were observed at 10.87 and 10.92 GHz, which originate from the SMF-1 and the MCF, respectively. The BGS change when strain was applied only to the MCF is also shown in Fig. 3(a). With increasing strain, the BGS of the SMF-1 hardly changed, while the BGS of the MCF shifted to higher frequency. As the small peaks observed at 10.95 and 11.03 GHz did not exhibit strain dependence, they are the second- and third-order Brillouin peaks of the SMF-1, respectively^16^,^19^. The instability of the peak power of the MCF was caused by the spectral overlap with the higher-order peaks of the SMF-1 and by the polarization-dependent fluctuations. Using this result, we plotted the BFS of the central core of the MCF as a function of strain (Fig. 3(b)). The dependence was almost linear with a proportionality constant of 484.8 MHz/% (with an error of ±~5 MHz/%; calculated from repetitive measurements), which is in agreement with the value (~493 MHz/%) in a standard silica SMF at 1.55 μm^17^.

**Figure 4:**
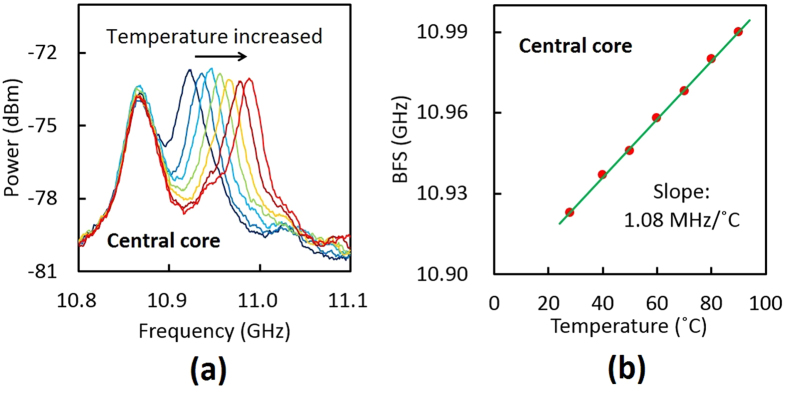
(**a**) BGS dependence on temperature (28, 40, 50, 60, 70, 80, 90 °C) and (**b**) BFS dependence on temperature in the central core of the MCF.

We then measured the BGS dependence on temperature in the central core of the MCF (Fig. 4(a)). With increasing temperature, only the BGS of the MCF shifted to higher frequency. Figure 4(b) shows the BFS dependence on temperature, which was also linear with a proportionality constant of 1.08 MHz/°C (with a measurement error of ±~0.03 MHz/°C). This value was almost the same as that (1.00 MHz/°C) in a standard silica SMF at 1.55 μm^18^.

### Outer-core characterization

**Figure 5:**
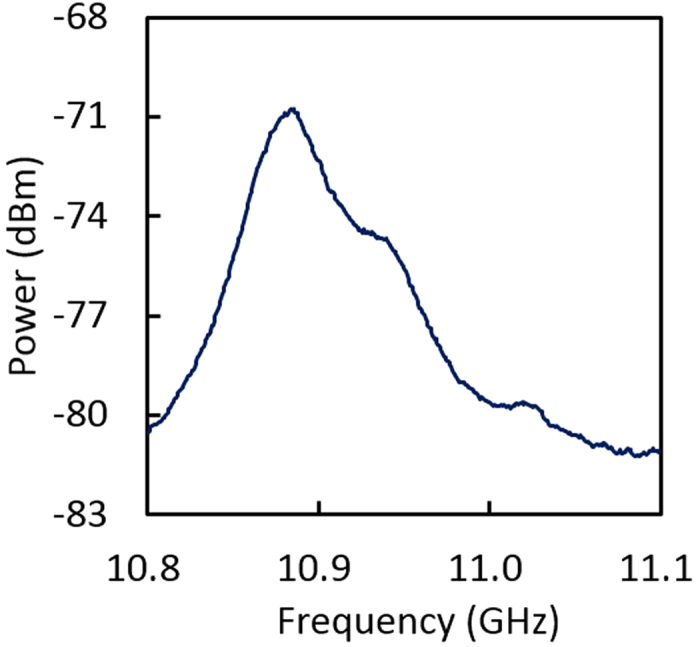
Measured BGS in the outer core of the MCF, overlapped with those in the SMF-1 and SMF-2.

Next, we present the measured results on the outer core of the MCF. The BGS measured at room temperature is shown in Fig. 5, where the Brillouin peak of the MCF was not clearly detected, because it overlapped with not only the peaks of the SMF-1 but also the peaks of the SMF-2 (including their higher-order peaks). To resolve this problem, we employed a differential measurement technique, with which the BGS of the SMF-1 and SMF-2 can be removed from the measured spectrum. The procedure was as follows: (1) obtain the BGS when local bending was applied to the SMF-2 at the region around the MCF/SMF-2 interface to induce considerable loss of over 40 dB; (2) obtain the BGS after the bending was released; and (3) subtract the BGS obtained in (1) from that obtained in (2) in log units. With this procedure, slight spectral distortion might be induced but the BFS values can be more accurately measured than without subtraction. Note that the region to which bending was applied was so short (<1 cm) that its influence on the measured results was negligible.

**Figure 6:**
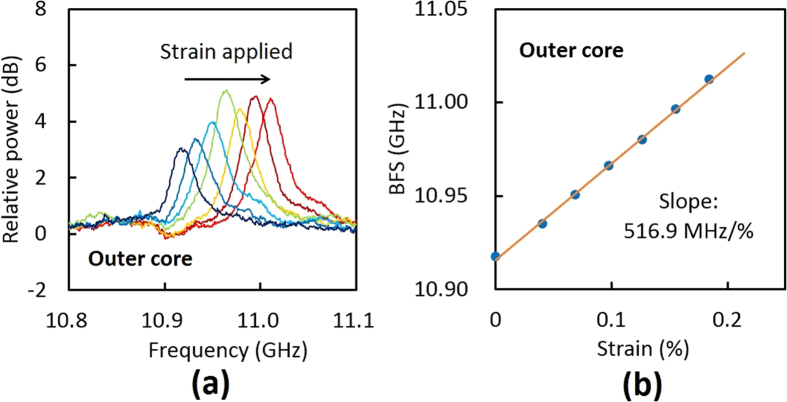
(**a**) BGS dependence on strain (0, 0.040, 0.069, 0.098, 0.126, 0.156, 0.184%) and (**b**) BFS dependence on strain in the outer core of the MCF.

The BGS measured at room temperature using the differential measurement technique is shown as the blue curve in Fig. 6(a). The BGS of the MCF was detected separately from those of the SMF-1 and SMF-2. The BFS of the outer core of the MCF was ~10.92 GHz, which is almost the same as that of the central core. These values are higher than any other reported value in a standard silica SMF by >50 MHz, indicating that the cores of this MCF have slightly higher degrees of hardness, and thus slightly higher acoustic velocities. This could also be explained as follows: the MFD of the MCF is 11.2 μm, whereas that of a standard silica SMF (G.652 series) is 10.5 μm at 1.55 μm. This indicates that the cores of the MCF are less doped with GeO_2_ than that of a standard silica SMF. Considering that the BFS decreases with increasing GeO_2_ doping concentration^42^, it is natural that higher BFS should be obtained in the MCF.

Subsequently, we measured the BGS dependence on strain in the outer core of the MCF, as also shown in Fig. 6(a). With increasing strain, the BGS of the MCF shifted to higher frequency. A dip independent of strain was generated at ~10.91 GHz by the differential measurement technique; along with the polarization-dependent fluctuations, this caused the instability of the peak power. The BFS dependence on strain in the outer core of the MCF is shown in Fig. 6(b). The proportionality constant was 516.9 MHz/%, which is ~30 MHz/% larger than that of the central core (484.8 MHz/%). This difference is not due to the measurement error (±~5 MHz/%). An extensive analysis on its origin is provided in the following section.

**Figure 7:**
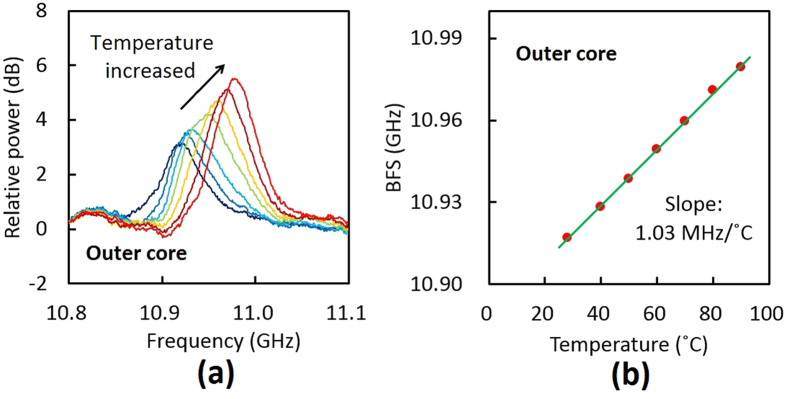
(**a**) BGS dependence on temperature (28, 40, 50, 60, 70, 80, 90 °C) and (**b**) BFS dependence on temperature in the outer core of the MCF.

The BGS dependence on temperature in the outer core of the MCF is also shown in Fig. 7(a). With increasing temperature, the BGS of the MCF shifted to higher frequency. From the temperature dependence of the BFS (Fig. 7(b)), the proportionality constant was calculated to be 1.03 MHz/°C, which was almost the same as those in the central core (1.08 MHz/°C) and a standard silica SMF (1.00 MHz/°C) at 1.55 μm^18^.

## Discussion

We discuss whether the observed difference in the BFS dependence between the central core and the outer core is caused by their structural or material difference. To investigate the difference in structural deformation when strain is applied to the MCF, the stress distribution in the cross-sectional direction was calculated using a commercial FEA software, ANSYS 11.0 (ANSYS Inc.). A real-size cross section of the MCF was modeled. For core material, the Young’s modulus *E* of 71 GPa, Poisson’s ratio *ν* of 0.166, and mass density *ρ* of 2220 kg/m^3^ were used, whereas for cladding material, *E* of 73 GPa, *ν* of 0.167, and *ρ* of 2220 kg/m^3^ were used (all these values were extracted from Figs 2 and 3 of Ref. 43). A longitudinal strain of 0.2% was uniformly applied.

Before presenting the simulation result, we here provide concrete evidence for the fact that the non-uniform strain distribution on the cross-section of the MCF is not the main reason. Suppose that the >5% discrepancy (observed in experiment) originates from the non-uniform strain distribution on the cross-section, and that, when 0.2% strain is applied to a 3-m-long MCF (actual measurement condition; see Figs 3(b) and 6(b)), the length of the central core has become 3.006 m. Then, the length of the outer core needs to be >3.0063 m, which means that one of the two strain-applied points should have a longitudinal displacement of >150 μm (=300 μm/2) between the central and outer cores. This situation is highly unlikely if we consider the core pitch of ~56 μm. Therefore, the non-uniform strain distribution on the cross-section is not the main reason for the discrepancy of the strain coefficients, and it is valid to assume that a longitudinal strain is uniformly distributed on the cross-section of the MCF.

**Figure 8:**
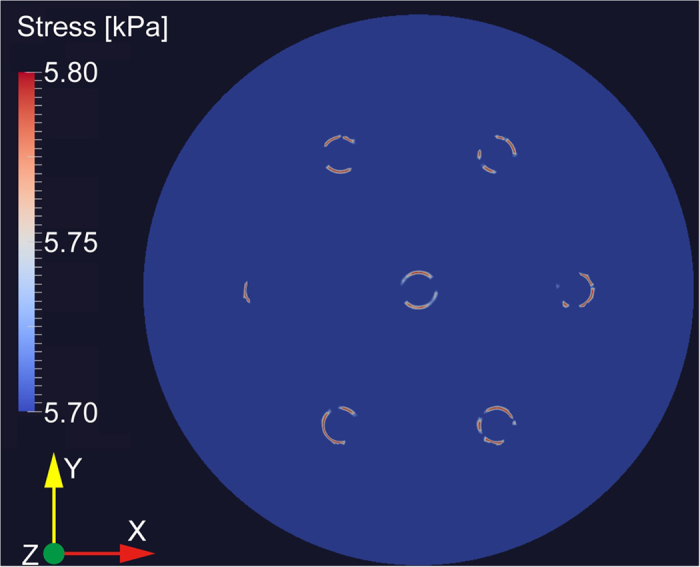
Simulated stress distribution in the cross-sectional direction.

The calculated stress distribution in the cross-sectional direction is shown in Fig. 8. The asymmetric nature seems to be caused by the meshing algorithm of the software. The stress was relatively high on the circumferences of the cores, and the stress applied to the outer cores (especially on their outer sides; indicated in red) turned out to be higher than that applied to the central core. However, even the highest stress obtained in the cross-sectional direction was approximately 6.2 kPa, which is negligibly low compared to the calculated longitudinal stress of ~14 MPa. Thus, we speculate that the difference in structural deformation between the central and outer cores cannot explain the difference in the BFS dependence on strain, which probably originates from the practical difference in core material (detailed information is not available).

In summary, we measured the BFS and its dependence on strain and temperature in the central core and one of the outer cores of a 7-core MCF. Although the strain dependence between the two cores was not perfectly identical, the strain- and temperature-dependence coefficients differed only slightly, which is to be expected because the MCF used in this measurement was fabricated with the intention that all the cores should be identical in material and structure. However, the difference in the Brillouin properties among the cores is expected to be enhanced by fabricating an MCF using different materials (see Table 1) and structures (note that several schemes have been developed to suppress the inter-core crosstalk by inducing phase mismatch among the cores)^12^,^13^,^14^,^15^. If this difference is sufficiently large, by exploiting Brillouin scattering in multiple cores of the MCF, a discriminative measurement of strain and temperature distributions will be feasible only by use of a single fiber^38^,^39^,^40^,^41^. Another potential application of Brillouin scattering in MCFs is bending/torsion sensing, because each core experiences different strains when bending or torsion is applied to the fiber. We anticipate that this paper will be an important archive exploring a new field of research: MCF Brillouin sensing.

## Additional Information

**How to cite this article**: Mizuno, Y. *et al*. Brillouin scattering in multi-core optical fibers for sensing applications. *Sci. Rep*. **5**, 11388; doi: 10.1038/srep11388 (2015).

## Figures and Tables

**Table 1:** BFS at Room Temperature and its Strain and Temperature Coefficients in Silica SMF, Tellurite Fibers, Chalcogenide Fibers, Bismuth-Oxide Fibers, Germanium-Doped PCFs, Erbium-Doped Fibers, Neodymium-Doped Fibers, Thulium-Doped Fibers, and PFGI-POFs at 1.55 μm.

Fiber	BFS (GHz)	*n*	Strain coefficient (MHz/%)	Temperature coefficient (MHz/K)
Silica SMF^a^	~10.85	~1.46	+580	+1.18
Tellurite^b^	~7.95	~2.03	–230	–1.14
Chalcogenide^c^	~7.95	~2.81	–	–
Bismuth-oxide^d^	~8.83	~2.22	–	−0.88
Ge-doped PCF^e^	~10.29	~1.46	+409	+0.82
Er-doped^f^	~11.42	~1.46	+479	+0.87
Nd-doped^g^	~10.82	~1.46	+466	+0.73
Tm-doped^g^	~10.90	~1.46	+433	+0.90
PFGI-POF^h^	~2.83	~1.35	–122	–4.09

The Refractive Index n of Each Fiber is Also Presented.

^a^Refs 17,18.

^b^Refs 25,26.

^c^Ref 27.

^d^Refs 26,29.

^e^Ref 30.

^f^Ref 31.

^g^Ref 32.

^h^Ref 35.
